# Lymph nodes as barriers to T‐cell rejuvenation in aging mice and nonhuman primates

**DOI:** 10.1111/acel.12865

**Published:** 2018-11-14

**Authors:** Heather L. Thompson, Megan J. Smithey, Jennifer L. Uhrlaub, Ilija Jeftić, Mladen Jergović, Sarah E. White, Noreen Currier, Anna M. Lang, Afam Okoye, Byung Park, Louis J. Picker, Charles D. Surh, Janko Nikolich‐Žugich

**Affiliations:** ^1^ Department of Immunobiology University of Arizona College of Medicine‐Tucson Tucson Arizona; ^2^ Arizona Center on Aging University of Arizona College of Medicine‐Tucson Tucson Arizona; ^3^ Honors College University of Arizona Tucson Arizona; ^4^ Vaccine and Gene Therapy Institute Oregon Health and Science University Beaverton Oregon; ^5^ Oregon National Primate Research Center Beaverton Oregon; ^6^ Knight Cancer Center Oregon Health and Science University Portland Oregon; ^7^ Academy of Immunology and Microbiology Institute for Basic Science Pohang South Korea; ^8^ Department of Integrative Biosciences and Biotechnology Pohang University of Science and Technology Pohang South Korea; ^9^ Division of Developmental Immunology La Jolla Institute for Allergy and Immunology California; ^10^ Present address: Division of Allergy, Pulmonary and Critical Care Medicine, Department of Medicine University of Wisconsin School of Medicine and Public Health Madison Wisconsin

## Abstract

In youth, thymic involution curtails production of new naïve T cells, placing the onus of T‐cell maintenance upon secondary lymphoid organs (SLO). This peripheral maintenance preserves the size of the T‐cell pool for much of the lifespan, but wanes in the last third of life, leading to a dearth of naïve T cells in blood and SLO, and contributing to suboptimal immune defense. Both keratinocyte growth factor (KGF) and sex steroid ablation (SSA) have been shown to transiently increase the size and cellularity of the old thymus. It is less clear whether this increase can improve protection of old animals from infectious challenge. Here, we directly measured the extent to which thymic rejuvenation benefits the peripheral T‐cell compartment of old mice and nonhuman primates. Following treatment of old animals with either KGF or SSA, we observed robust rejuvenation of thymic size and cellularity, and, in a reporter mouse model, an increase in recent thymic emigrants (RTE) in the blood. However, few RTE were found in the spleen and even fewer in the lymph nodes, and SSA‐treated mice showed no improvement in immune defense against West Nile virus. In parallel, we found increased disorganization and fibrosis in old LN of both mice and nonhuman primates. These results suggest that SLO defects with aging can negate the effects of successful thymic rejuvenation in immune defense.

**Abbreviations**
A: adult
KGF: keratinocyte growth factor
LN: lymph nodes
O: old
RTE: recent thymic emigrants
SLO: secondary lymphoid organs
SSA: sex steroid ablation

## 1 INTRODUCTION

The thymus undergoes age‐related involution, that includes progressive loss of thymic epithelial and hematopoietic lineage cellularity, an increase in adiposity, and reduced T‐cell output (Hale, Boursalian, Turk, & Fink, 2006; Nikolich‐Žugich, 2014). In the periphery, fewer naïve T cells are available (Appay & Sauce, 2014), and the old T‐cell compartment is less able to respond to infections and cancer. This is believed to contribute to increased vulnerability of older adults to emerging and reemerging infections (Nikolich‐Žugich, 2014). More recent evidence suggests that secondary lymphoid organ (SLO) organization and structure also undergo changes with increased age (Aw et al., 2016; Becklund et al., 2016; Davies, Thompson, Pulko, Padilla Torres, & Nikolich‐Žugich, 2017; Thompson, Smithey, Surh, & Nikolich‐Žugich, 2017), and the impact of these changes upon naïve T‐cell survival (Link et al., 2007) and function is beginning to be understood.

A “holy grail” of T‐cell aging research is to achieve functional rejuvenation of T‐cell function (Nikolich‐Žugich, 2018). Early experiments with surgical castration have shown that transient thymic rejuvenation is possible, as measured by increased thymic volume and cellularity (Fitzpatrick, Kendall, Wheeler, Adcock, & Greenstein, 1985). Similar results have since been obtained using pharmacological sex steroid blockade as well as injection of growth factors (Heng et al., 2005; Min et al., 2007; Velardi et al., 2014). While some of these studies have shown some improvement in peripheral immune function in treated mice (Heng et al., 2012; Min et al., 2007), the ultimate tests of functional immunity in the face of microbial challenge were not performed. Therefore, the question remains how well thymic rejuvenation improves the peripheral T‐cell pool with aging, and whether it confers improved protection against infection.

To address this question, we examined the effects of (a) keratinocyte growth factor (KGF) administration in mice and nonhuman primates, or (b) sex steroid ablation (SSA) in mice using an antagonist of the luteinizing hormone‐releasing hormone receptor, degarelix (Firmagon). Despite robust thymic rejuvenation in response to both interventions, we found no evidence of improved peripheral T‐cell maintenance. KGF‐treated old mice were not more effective at mounting CD8 T‐cell responses to, or clearance of, *Listeria monocytogenes*. Similarly, degarelix did not improve CD8 T‐cell responses to, or survival of old mice following challenge with, West Nile virus (WNV). While rejuvenated thymi produced substantial numbers of recent thymic emigrants (RTE), these RTE did not significantly contribute to T‐cell populations in the SLO of old mice compared to adults. We further found that old lymph nodes exhibited considerable fibrosis and degeneration of structure. These data indicate that restoration of thymic function by itself may not be sufficient to improve the immune response in elderly and suggest that interventions to simultaneously alleviate defects in aging SLO may need to be considered when designing strategies to improve immune response in older organisms.

## 2 RESULTS

### 2.1 Age‐related decline in naïve T cells

**Figure 1:**
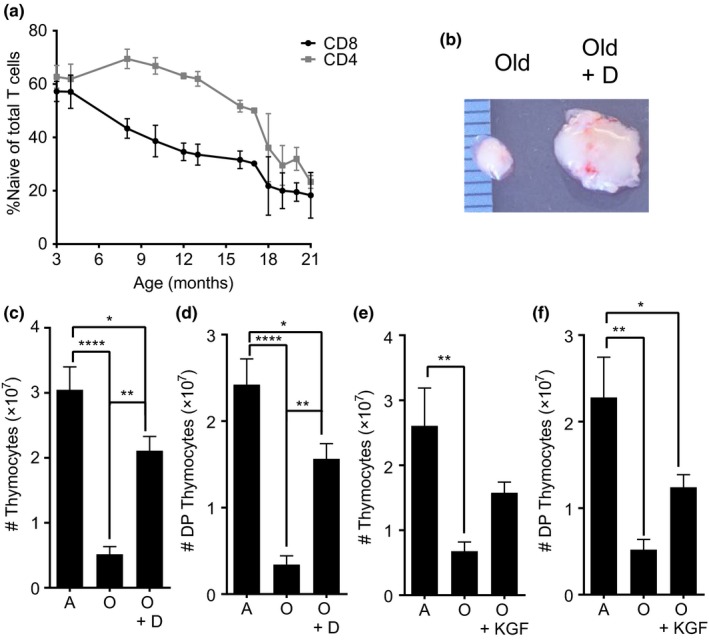
Thymic rejuvenation increases thymocyte populations in old mice. (a) Representation of naïve T cells among total CD4 (gray) or total CD8 (black) pool from mice across the lifespan is shown. Cells were gated on CD4+ or CD8+ cells, and then on CD62^HI^ CD44^LO^. The slopes denote a decline from youth, and are significantly different from zero, *p* < 0.0001 for CD8 and *p* = 0.0003 for CD4. Data represent *n* = 81 mice of indicated ages across the timeline. (b–f) Mice were treated with either degarelix (D) or KGF and analyzed 42 days or 1 month post‐treatment, respectively. (b) Thymus from an untreated old mouse (left) and a degarelix‐treated old mouse (right). (c) Thymocyte numbers from adult, old, and old mice treated with degarelix. (d) Double‐positive thymocytes from adult, old, and old mice treated with degarelix. (e) Thymocyte numbers from adult, old, and old mice treated with KGF. (f) Numbers of double‐positive thymocytes from adult, old, and old mice treated with KGF. For degarelix experiments, *n* = 7 to 13 mice per group pooled from two independent experiments. For KGF experiments, *n* = 3–7 mice per group. Means + *SEM* are shown (*p < 0.05, **p < 0.01, ***p < 0.001, ****p < 0.0001)

Early in life, thymic involution progressively limits the production of new naïve T cells (Nikolich‐Žugich, 2014). Thereafter, the naïve T‐cell pool is successfully maintained peripherally until the last tertile or quartile of life (den Braber et al., 2012), at which point this process also eventually deteriorates. This leads to a loss of naïve T cells that is concordant with an increase in susceptibility to infection with advanced age (Heng et al., 2012; Nikolich‐Žugich, 2014). Figure 1a shows the cross‐sectional kinetics of naïve CD8 and CD4 T cells (CD62L^HI^, CD44^LO^) decline in our mouse colony, measured as a fraction of total CD8 and CD4 cells, respectively. We have previously shown a similar loss in the absolute number of naïve CD8 T cells with age in the mouse spleen (Smithey, Li, Venturi, Davenport, & Nikolich‐Žugich, 2012). The decline in representation and/or numbers of naïve CD8 T cells in the blood had also been described by several groups in nonhuman primates (Janković, Messaoudi, & Nikolich‐Žugich, 2003; Okoye et al., 2015; Pitcher et al., 2002) and humans (Fagnoni et al., 2000; Olsson et al., 2001; Wertheimer et al., 2014).

### 2.2 SSA or KGF treatment improves thymic cellularity in aged mice

Thymic rejuvenation has been demonstrated in the old age (Fitzpatrick et al., 1985; Heng et al., 2005; Sutherland et al., 2005), and KGF and SSA have been shown to improve thymic cellularity in middle‐aged (KGF) and old mice (SSA) (Heng et al., 2012; Min et al., 2007; Velardi et al., 2014). We used mice >18 months old, based on the NIA definition of old mice (Miller & Nadon, 2000), and the doses of KGF and degarelix reported to increase thymic cellularity and size (Min et al., 2007, 2002 ; Seggewiss et al., 2007) and confirmed the above results. Thus, degarelix induced a robust increase in thymic size compared to untreated controls (Figure 1b), with a fourfold increase in the number of thymocytes (from 5.17 × 10^6^ ± 1.16 × 10^6^ in old controls to 2.11 × 10^7^ ± 2.19 × 10^6^ 42 days post‐treatment) (Figure 1c). The number of CD8/CD4 double‐positive (DP) thymocytes has been used as a measure of thymic generative activity, because the ratio of DP cells to RTE remains relatively constant with age (Hale et al., 2006). Degarelix boosted the numbers of double‐positive thymocytes in old animals by 4.6‐fold (Figure 1d) from 3.42 × 10^6^ ± 1.01 × 10^6^ (old controls) to 1.56 × 10^7^ ± 1.77 × 10^6^ (SSA). KGF also increased total thymocyte numbers by 2.3‐fold from 6.77 × 10^6^ ± 1.42 × 10^6^ (old untreated) to 1.58 × 10^7^ ± 1.65 × 10^6^, but this difference did not reach statistical significance, most likely due to low power (Figure 1e). Similarly, KGF treatment increased the number of DP thymocytes by 2.4‐fold from 5.21 × 10^6^ ± 1.18 × 10^6^ (old untreated) to 1.24 × 10^7^ ± 1.45 × 10^6^ (Figure 1f). This increase, also, did not reach statistical significance.

### 2.3 KGF or SSA DO not increase naïve CD8 or CD4 T‐cell numbers in blood

**Figure 2:**
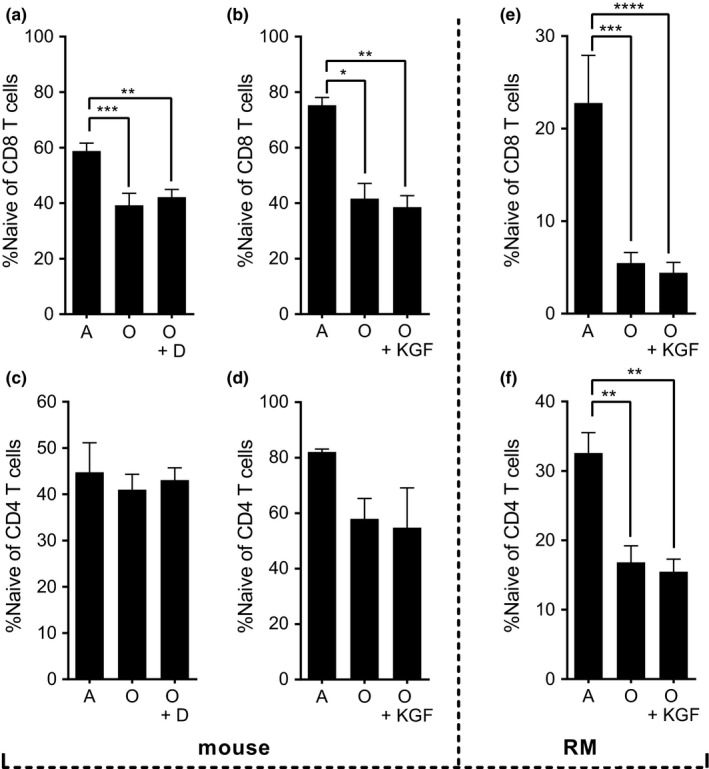
Thymic rejuvenation does not improve the frequency of naïve CD8 in the blood. (a) Percentage of CD8 T cells with naïve phenotype (CD62^HI^ CD44^LO^) in the blood of adult, old, and old mice treated with degarelix (D) for 42 days. (b) Percentage of CD8 T cells with naïve phenotype in the blood of adult, old, and old mice treated with KGF at 1 month post‐treatment. (c) Percentage of CD4 T cells with naïve phenotype (CD62^HI^ CD44^LO^) in the blood of adult, old, and old mice treated with degarelix for 42 days. (d) Percentage of CD4 T cells with naïve phenotype in the blood of adult, old, and old mice treated with KGF at 1 month post‐treatment. (e) Percentage of naïve T cells (CD95^LO^, CD28^MOD^) in the CD8 T‐cell pool of RM adult, old, and KGF‐treated old at 21 days post‐treatment. (f) Percentage of naïve T cells (CD95^LO^, CD28^MOD^) in the CD4 T‐cell pool of RM adult, old, and KGF‐treated old at 21 days post‐treatment. For RM experiments, *n* = 3 adults, *n* = 7 old, and *n* = 7 treated old from three independent experiments. For degarelix experiments, *n* = 7 to 13 mice per group pooled from two independent experiments. Bar graphs means + *SEM* are shown (*p < 0.05, **p < 0.01, ***p < 0.001, ****p < 0.0001). Absolute numbers provided in the text

We next examined whether increased thymic size and cellularity contributed to the peripheral naïve T‐cell pool in the blood and found that the increase in thymus cellularity failed to increase the percentage of naïve CD8 (Figure 2a,b) or naïve CD4 (Figure 2c,d) T cells in the peripheral blood of old mice following either degarelix (Figure 2a,c) or KGF (Figure 2b,d) treatment. Because the absolute numbers of CD3 T cells in blood tend to decrease with age (e.g., # CD3/ml blood 1.99 ± 0.43 × 10^6^ vs. 1.03 ± 0.27 × 10^6^; *p* = 0.0159), these results are even more pronounced in absolute terms. Similarly, old rhesus macaques (RM) treated with KGF showed no statistical improvement in the percentage of naïve CD8 or CD4 T cells (Figure 2e,f) (defined, as previously described (Okoye et al., 2015), as CD28^INT^CD95^LO^) in blood. We could not directly measure thymic cellularity in RM treated with KGF as that would have been a terminal study, and our attempts to measure thymic size by MRI were not satisfactory. However, we did observe comparable clinical signs of KGF activity in old and adult RM, as manifested by transient redness/flushing of the face and lips and increased salivation, which suggested that the administered KGF had the expected impact on epithelia. Moreover, the same dose (in mg/kg) produced a manifest increase in murine thymus cellularity. We conclude that thymic rejuvenation via SSA or KGF administration did not correlate to an increased frequency of naïve peripheral T cells in SLO.

### 2.4 SSA treatment of old mice did not improve survival after WNV challenge

**Figure 3:**
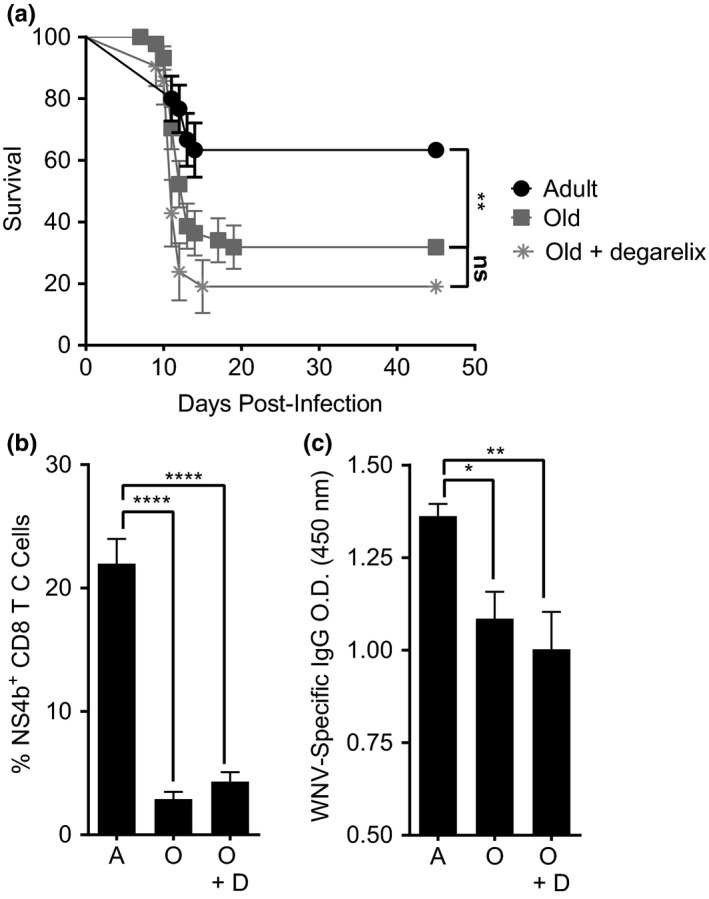
Thymic rejuvenation is insufficient to protect against lethal WNV infection. (a) Survival of adult, old, and old mice treated with degarelix for 42 days then challenged with WNV *n* = 20–50 mice per group pooled from two independent experiments. (b) Percent of H‐2D^b^/NS4b_2488_ tetramer cells in the blood of adult, old, old treated with degarelix‐treated mice at day 7 following infection *n* = 5 mice per group, one experiment showed representative of two experiments. (c) WNV‐specific IgG at day 7 following infection, *n* = 10 mice per group, one experiment showed representative of two experiments. For bar graphs, means + SEM are shown (*p < 0.05, **p < 0.01, ***p < 0.001, ****p < 0.0001)

While in our hands the increased thymic size and cellularity did not translate into an overall increase in naïve T‐cell frequency in the blood, it was possible that the newly produced “young” cells replaced older naïve T cells. This would be consistent with prior data from the Fink group, showing the replacement of existing naïve T cells in the spleen with newly produced RTE (Hale et al., 2006). Such replacement of “old” naïve T cells could lead to improved immunity and was invoked by Haynes, Swain, and colleagues to explain improved T‐cell function following depletion of peripheral T cells in old mice (Haynes, Eaton, Burns, Randall, & Swain, 2005). To test whether the T‐cell pool was functionally improved by SSA, we challenged old degarelix‐treated and control mice with WNV 42 days after SSA. WNV is an age‐sensitive virus that induces significantly higher mortality rates in older humans (Petersen & Marfin, 2002) and mice (Brien, Uhrlaub, Hirsch, Wiley, & Nikolich‐Žugich, 2009) and is ideal to test possible improvements in protective immunity of older organisms. We found that the survival of old mice treated with degarelix was no better, and tended to be worse, compared to untreated controls (Figure 3a). However, as the defense against WNV is mediated by multiple arms of immunity (Suthar, Diamond, & Gale, 2013), it was possible that T‐cell responses could have been improved regardless of similar mortality. To address this possibility, we measured the fraction of WNV NS4b‐specific CD8+ T cells in the blood using the H‐2D^b^/NS4b_2488_ tetramer (Uhrlaub, Brien, Widman, Mason, & Nikolich‐Zugich, 2011) and found no improvement in this response in the blood at day 7 (peak of the response) between degarelix‐treated and control old mice (Figure 3b). We have also performed several analogous experiments with KGF in old mice infected for life with herpesviruses (HSV‐1 and CMV, used to mimic human exposure to herpesviruses). Following challenge with *Listeria monocytogenes* carrying recombinant ovalbumin (Lm‐OVA; protection is mediated by CD8 T cells), we found no improvement in CD8 responses in KGF‐treated old mice over old untreated controls (regardless of herpesvirus infection). In fact, there was a trend toward lower total levels of CD8 T cells specific for OVA (not shown), as well as in their polyfunctionality (Supporting Information Figure S1).

Prior work with KGF treatment of 14‐month‐old mice found improved anti‐KLH antibody responses and took it as evidence that CD4 function must have been improved (Min et al., 2007). To assess that possibility, we examined anti‐WNV antibody responses. Adult mice exhibited significantly higher overall anti‐WNV IgG than old mice, and degarelix again provided no advantage over old controls in that regard (Figure 3c). Together, these results indicate that thymic rejuvenation using degarelix or KGF was insufficient to improve immunity against intracellular infections in old mice.

### 2.5 Increased thymic output does not increase naïve T cells in secondary lymphoid organs

**Figure 4:**
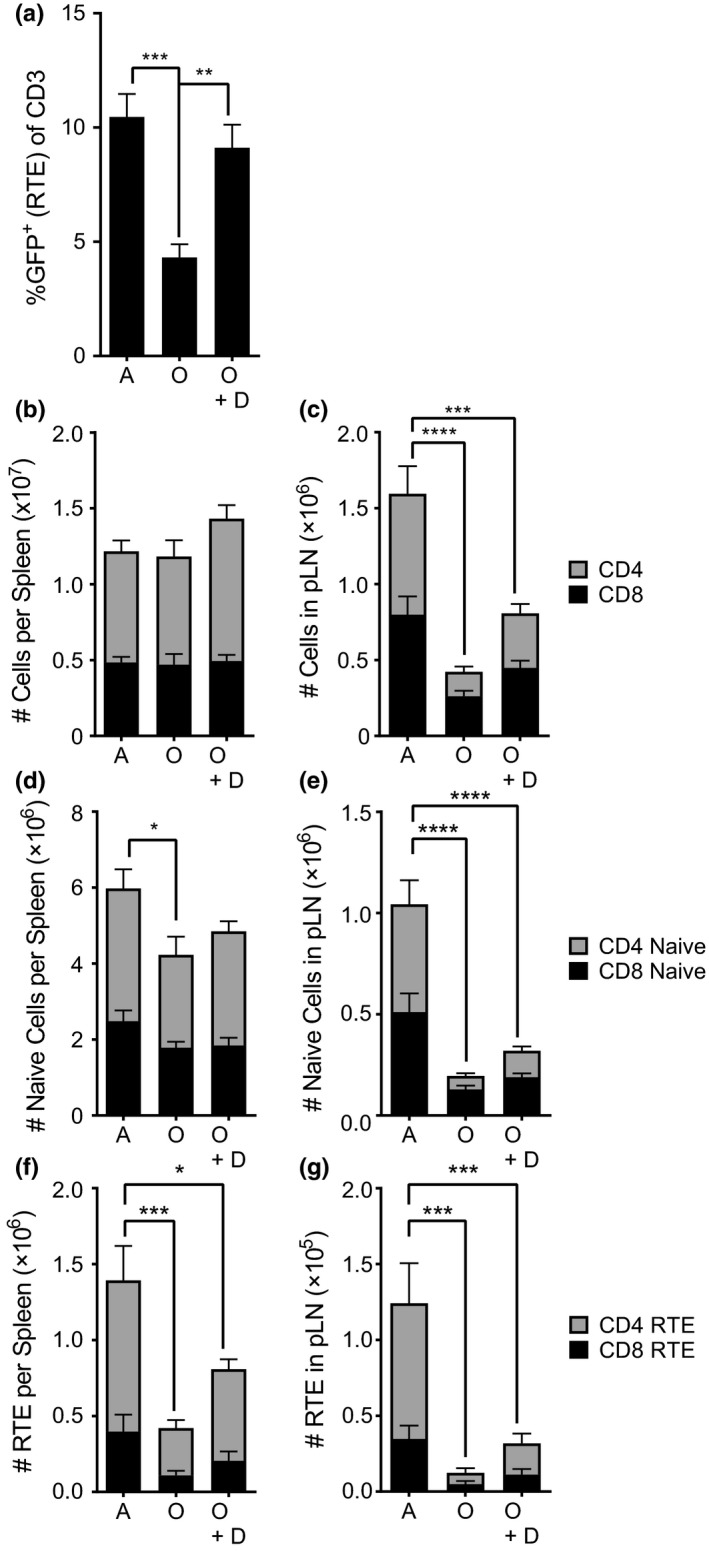
Secondary lymphoid compartments are not restored by recent thymic emigrants following thymic rejuvenation. (a) Frequency of total T cells that are recent thymic emigrants in the blood of adult, old, and old mice treated with degarelix (D). (b) CD4 (gray) and CD8 (black) T cells present in spleens of adult, old, and old mice treated with degarelix (D). (c) CD4 (gray) and CD8 (black) T cells present in the peripheral lymph nodes (pool of one of each inguinal, popliteal, and brachial). (d) Naïve T cells in the spleen of adult, old, and old mice treated with degarelix. (e) CD4 (gray) and CD8 (black) naïve T cells in the peripheral lymph nodes of adult, old, and old mice treated with degarelix (D). (f) CD4 (gray) and CD8 (black) recent thymic emigrants present in the spleen of adult, old, and old mice treated with degarelix (D). (g) CD4 (gray) and CD8 (black) recent thymic emigrants in the peripheral lymph node pool of adult, old, and old + degarelix (D). *n* = 7 to 13 mice per group, results are pooled from two independent experiments. Statistics were done on pooled CD4+ and CD8+ for each population shown. Means + SEM are shown (*p < 0.05, **p < 0.01, ***p < 0.001, ****p < 0.0001). Statistics were done on the total bar

To examine the mechanistic basis of the failure of the rejuvenated thymus to improve functional immunity, we took advantage of Rag2pGFP transgenic mice, in which the RTE are labeled with GFP, and where this label persists in newly exported cells until diluted by cell division and/or until the cell is removed from the population (Boursalian, Golob, Soper, Cooper, & Fink, 2004; Hale et al., 2006). The Rag2pGFP reporter marks RTE for approximately 3 weeks following thymic export (Boursalian et al., 2004) and is therefore a robust marker of RTE production and initial distribution. In this model, we found a 2.12‐fold increase in the percentage of CD3‐expressing RTE in the blood in old mice on day 42 following degarelix (Figure 4a), at the time when the total fraction of naïve T cells in the blood remained unaltered (Figure 2). This result has been seen at other time points (Figure 2, day 30 post‐KGF; not shown—day 14 postdegarelix and day 60 post‐KGF), ruling out the possibility that we have simply missed the time point where the increase in naïve T cells was evident. This suggests that SSA mediates the increased RTE output from the thymus, but that this increase is insufficient to increase the total fraction of naïve T cells in the blood.

Changes in the stromal microenvironment in secondary lymphoid organs (SLO), particularly lymph nodes (LN), have recently been reported to influence naïve T‐cell homeostasis (Becklund et al., 2016; Link et al., 2007). We found that old LN exhibit a profound decline in T‐cell cellularity while the total T‐cell numbers in the spleen were not significantly altered in old mice (Figure 4b–c). LN in old animals contained 3.4‐fold fewer T cells than adults (Figure 4c), consistent with previous reports (Davies et al., 2017). Degarelix treatment improved the number of total T cells compared to old untreated mice, but the T‐cell numbers in treated animals remained significantly lower (1.9‐fold compared to adults, Figure 4c). The numbers of naïve T (CD4 plus CD8) cells were also significantly reduced in the spleen (a 1.4‐fold reduction compared to the adult, Figure 4d), and even more remarkably, in the LNs (4.9‐fold vs. adult controls, Figure 4e), and, despite some trending, SSA did not significantly improve their numbers compared to untreated controls (Figure 4d–e, unless indicated, no *p*‐values less than 0.1 by ANOVA). Finally, we examined the contribution of naïve RTE to the naïve T‐cell pool in SLO using adult and old RAG2pGFP mice, and found that naïve RTEs (CD62L^HI^CD44^LO^, GFP^+^) were drastically decreased in both old spleen and LN compared to adult controls (Figure 4f–g, unless indicated, no *p*‐values less than 0.1 by ANOVA).

### 2.6 Increased thymic output contributes differently in adult and old mice to the peripheral T‐cell pool in Slo

**Figure 5:**
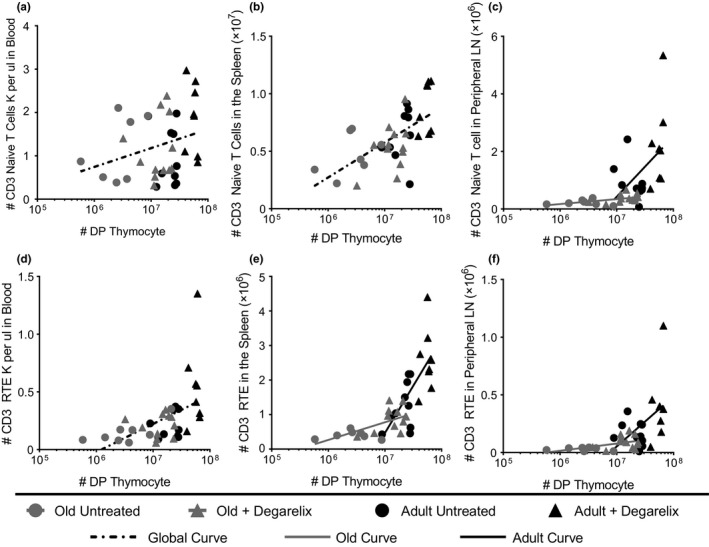
Thymic influence on naïve T‐cell pool in SLO is altered with age. (a) Double‐positive thymocytes compared to the number of CD3+ naïve T cells per ul of blood in old mice, old mice treated with degarelix, adult mice, and adult mice treated with degarelix, one curve fits both data sets. (b) Double‐positive thymocytes compared to the number of CD3+ naïve T cells per spleen, one curve fits both data sets. (c) Double‐positive thymocytes compared to CD3+ naïve T cells in peripheral lymph node pool, a different curve set for each adult compared to the old lymph node data set (*p* = 0.0142). (d) Number of blood CD3 RTE determined by Rag2pGFP reporter compared to the number of double‐positive cells in the thymus, one curve fits both data sets. (e) Number of spleen CD3 RTE compared to double‐positive thymocytes, different curve set is needed for old compared to adult data sets (*p* = 0.0011). (f) Number of lymph node CD3 RTE compared to double‐positive thymocytes, a different curve is needed to fit each data set (*p* = 0.0427). *n* = 19 mice per group, results are pooled from two independent experiments

Old mice exhibit defects in the stromal architecture of SLO (Aw et al., 2016; Becklund et al., 2016; Davies et al., 2017; Masters, Haynes, Su, & Palmer, 2016; Thompson et al., 2017) and that could play a role in the suboptimal ability of old LN to recruit and properly direct T‐cell trafficking during an immune response (Richner et al., 2015). Less is known about whether such defects could also affect ingress of RTE into old LN. To test whether the kinetics of thymic and peripheral (SLO) reconstitution may be different between adult and old mice, we treated old and adult mice with degarelix and measured numbers of double‐positive (DP) thymocytes (as a measure of thymic generative activity (Hale et al., 2006)) against the number of naïve CD3 T cells (CD3^+^CD62L^HI^CD44^LO^) in different lymphoid tissues. We found a linear relationship between the number of DP thymocytes and numbers of total naïve T cells in the blood and spleen regardless of age or treatment. Consequently, all dots could be fitted around a single line by linear regression (Figure 5a–b). That relationship, however, did not hold between DP thymocyte numbers and the numbers of naïve T cells in the LN, where many fewer naïve T cells were present, causing the two regression lines to differ significantly between adult and old mice (*p* = 0.0142; Figure 5c). To track how RTE were contributing to the naïve T‐cell pool during degarelix‐mediated rejuvenation, we used RAG2pGFP mice. DP thymocyte numbers directly and linearly correlated to the naïve CD3 RTE pool found in the adult and old blood (Figure 5d), suggesting that newly produced T cells migrate into blood with no restriction. However, the same was not observed for the old SLO (spleen, Figure 5e; LN, Figure 5f), where there were many fewer RTE than would have been predicted based on the number of thymic DP cells. Therefore, increasing thymic output with degarelix produced RTE that incorporated readily into adult, but not old, SLO (Figure 5e–f). We hypothesize that age‐related changes in the SLO are an additional barrier to immune rejuvenation in old animals that limits the beneficial effects of thymic rejuvenation.

### 2.7 Old LN exhibit pronounced fibrotic changes with aging

**Figure 6:**
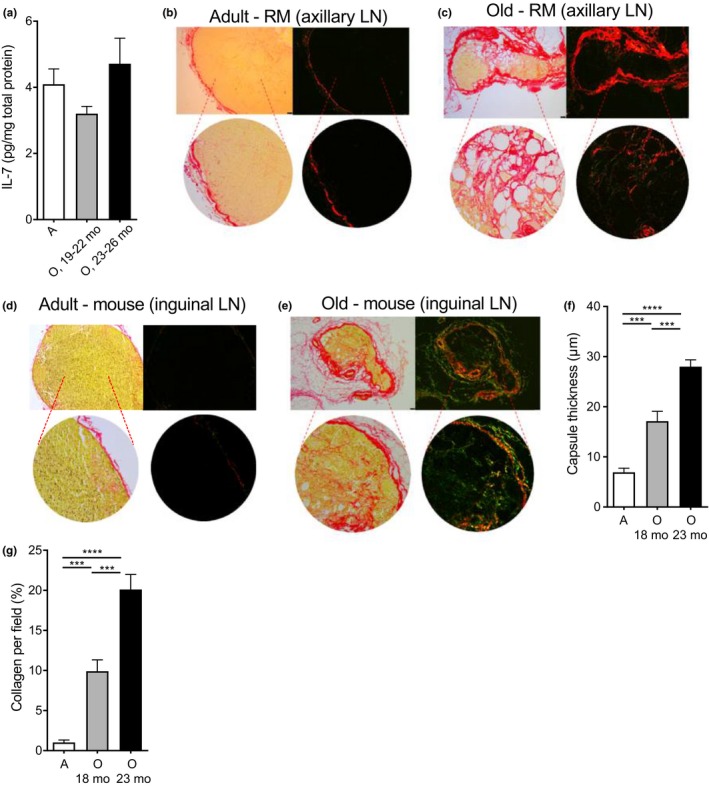
Old LN exhibit increased fibrosis in both mice and nonhuman primates. Photomicrographs show lymph node sections stained for collagen with Picrosirius red under bright field (left) or polarization contrast (right) (Leica, DMI6000). Representative images (one animal of four analyzed, similar changes observed in others) showing collagen content in (a). Total IL7 protein (pg/ml) as determined by ELISA on total LN homogenate for adult (A), old (O, 19–21 months), and old (O, 22–24 months) mice. (b) Adult rhesus macaque (axillary LN, 8 years old—scale bar, 100 μm); (c) old rhesus macaque (axillary LN, 25 years old—scale bar, 100 μm); (d) adult mice (inguinal LN, 4 months of age—scale bar, 100 μm); (e) old mice (inguinal LN, 23 months of age—scale bar, 100 μm). Fibrosis in mouse LNs was quantified using *ImageJ* software and presented as (f) capsule thickness and (g) percentage of area positive for collagen inside the capsule. Mean + *SEM* are shown (*p < 0.05, **p < 0.01, ***p < 0.001, ****p < 0.0001), *n* = 4–6 mouse/group)

LN are the main sites of naïve T‐cell maintenance, and the key cell in that maintenance is the fibroblastic reticular cell (FRC) (Link et al., 2007; Mueller & Ahmed, 2008). FRC secretes IL‐7 critical for naïve T‐cell maintenance and deposits it on the extracellular matrix (dominantly collagenous) that they themselves also produce. Both the extracellular matrix and the process of FRC branch to form the critical 3‐D meshwork of conduits that allow directional T‐cell migration and the delivery of homeostatic maintenance signals. We have recently shown in two separate and independent studies that numbers of FRC (Becklund et al., 2016; Davies et al., 2017) as well as of lymphoid endothelial cells (LEC) (Davies et al., 2017) diminish significantly in old LN, with the FRC network showing marked disorganization (Becklund et al., 2016). By contrast, we found no evidence that production of IL‐7 was reduced in old LN at the mRNA (Becklund et al., 2016) or protein (Figure 6a) levels.

This raised the possibility that bioavailability/access to IL‐7 may be the key mechanism behind the above inability of old LN to recruit and/or retain RTE. Indeed, in response to TGFβ and/or Th2 cytokines (particularly IL‐13), FRC has the potential to produce excessive collagen, and to lead to the process of fibrosis, that results in a thickened, sclerotic capsule, disorganized LN areas and an accumulation of thick collagen fibers in the LN parenchyma (Fletcher, Acton, & Knoblich, 2015). To assess whether fibrotic changes may occur in the aged LN, we stained LN of old and adult RM and mice with *Picrosirius Red* (Figure 6b–e). This allowed visualization and quantification of collagen, with the intensity of the red signal being proportional to the thickness of collagen (Figure 6f). LNs in both RM and mice exhibited pronounced signs of fibrosis with age, manifested as outer capsule thickening, overall LN size reduction, and stromal infiltration with thick collagen bundles, so that more than 10% of the fields in murine LN contained collagen fibers, as opposed to <2% for the adult animal (Figure 6f). Preliminary analysis of gene expression in old and adult LN was consistent with the above findings, with increased production of TGFβ and IL‐13 in old mice (I. Jeftic et al., in preparation), and experiments are in progress to conclusively assign such production to defined lymphocyte or stromal subsets in SLO. These results are consistent with impaired influx into and/or retention of RTE into, LN as a consequence of aging, although additional studies will be needed to fully establish mechanistic connection(s).

## 3 DISCUSSION

KGF and SSA have been shown to rejuvenate old thymus (Alpdogan et al., 2006; Chinn, Blackburn, Manley, & Sempowski, 2012; Heng et al., 2012; Velardi et al., 2014). Consistent with this, we found an improvement in thymocyte number following either KGF or SSA/degarelix treatment of old mice (Heng et al., 2012; Velardi et al., 2014) (Figure 1). However, while the thymi of old mice treated with degarelix or KGF were rejuvenated, we found no increase in naïve CD8 and CD4 T‐cell frequencies in the blood in mice or monkeys (Figure 2). We also found that thymic rejuvenation resulted in a surge of RTE in the blood, but that it did not significantly improve the number of naïve T cells or RTE in spleen or LN in old mice (Figures 4, 5). Even when RTE numbers were trending higher (but were not significantly improved), these numbers were too low (only 2%–10% of all total naïve T cells) to contribute to an increase in the naïve T‐cell pool or overall LN cellularity. This further suggested that LN were not capable of either recruiting and/or retaining new RTE produced by the thymus, an issue discussed further below.

It has been shown that a fraction of RTE can home directly to the gut and produce intraepithelial lymphocytes (Staton et al., 2006). We did not examine this migration, as our aim was to deal with immunity and homeostasis in SLO, and therefore, aging of mucosal lymphoid tissues will have to be addressed at a later date.

KGF is secreted by mesenchymal stem cells and is known to promote the proliferation of epithelial cells including thymic epithelial cells (Chaudhry, Velardi, Dudakov, & Brink, 2016; Nikolich‐Žugich, 2007). KGF is currently licensed for the reduction in oral mucositis after bone marrow transplantation (Seggewiss et al., 2007). KGF was shown to increase thymopoiesis and total T‐cell numbers in the spleen of aged mice (18 months old) due to an expansion of both naïve and memory subsets (Chaudhry et al., 2016). However, T‐cell proliferation in mixed lymphocyte reactions or following concanavalin A stimulation was not improved by KGF (Alpdogan et al., 2006). Min et al. treated 14‐month‐old mice with KGF and observed increased thymopoiesis and increased total CD4 and CD8 T cells in the blood 1 month after treatment, which correlated to improved secondary T‐dependent antibody responses to keyhole limpet hemocyanin (KLH) given in adjuvant (Min et al., 2007). There are several differences between these experiments and the ones described here that may explain the discrepancy of the results. First, we performed our experiments on *bona fide* old animals (18–20 months at treatment), as opposed to the “almost” old 14‐month‐old mice (Min et al., 2007). Second, it is possible that secondary KLH + adjuvant stimulation is a more potent stimulus compared to live WNV in our experiments; third, the experiments may not be directly comparable due to different rejuvenation reagents. Finally, there was a difference in the immunization route (s.c. for WNV following degarelix, and i.v. for *Listeria* following KGF in our hands; vs. i.p. for KLH + IFA in the experiments of Min et al. [2007]). At a minimum, protective immunity against *Listeria*, that is mediated by CD8 T cells, was not improved by any parameter tested (CD8 tetramer + cell numbers or CD8+ T‐cell polyfunctionality).

Sex steroid ablation has also been extensively studied for its ability to rejuvenate the aged thymus and the naïve T‐cell compartment (Chaudhry et al., 2016; Heng et al., 2012). SSA via surgical castration (Heng et al., 2012) or degarelix (Shore, 2012) can restore both thymic cellularity and the ratios of naïve to memory peripheral T cells in 9‐month‐old mice. However, surgical castration is progressively less effective in restoring peripheral naïve:memory T‐cell ratios in 18‐month‐old and 24‐month‐old mice (Heng et al., 2005), and degarelix has not been studied in that regard so far. More importantly, surgical SSA in 9‐month‐old mice led to improved virus‐specific CD8 T‐cell percentages and numbers following influenza A viral infection (Heng et al., 2012). That effect was somewhat reduced at 18 months and abrogated in 24‐month‐old mice (Heng et al., 2012). In our hands, degarelix improved thymic cellularity and the numbers of RTE in the peripheral blood of old (>18, typically 20 months) mice, but did not improve either total naïve or RTE numbers in their SLO. Further, degarelix treatment did not improve survival, or T‐ or B‐cell responses against WNV in old mice. Our functional data are reminiscent to data of Heng et al. (2012), in that they both highlight the existence of age limits to the ability of SSA to improve T‐ and B‐cell responses in truly old mice.

One key difference between our and the above studies is that we conclusively tracked RTE export and migration by rejuvenated thymi into the blood, spleen, and LN. Our finding of the reduced presence of RTE in SLO of old mice raises important issues about the exact mechanism of the age‐related defect in this case. Such a defect(s) could be intrinsic to newly produced T cells, or intrinsic to LN stroma, or both, and experiments are in progress to address this issue. Regardless, this defect results in impaired homing and/or retention in SLO of the aged animals.

While there is much to be learned about long‐term maintenance of stromal and lymphoid compartments in SLO with aging, we know that RTE need signals from SLO to survive and mature properly (Houston, Nechanitzky, & Fink, 2008; Link et al., 2007). Our data are consistent with recent work documenting a decline in FRC in old LN (Becklund et al., 2016) and showing that fewer transferred naïve T cells can be recovered from the old compared to adult SLO post‐WNV infection (Richner et al., 2015). Of interest, this study and a prior study of one of us (Becklund et al., 2016) have demonstrated that IL‐7 does not decline with age at the mRNA (Becklund et al., 2016) and protein (this study) levels, even though the FRC, a critical component of LN stroma, that produce IL‐7, is reduced with aging (Becklund et al., 2016; Davies et al., 2017). Together with our results demonstrating increased LN fibrosis with age, and our data, to be reported separately, on the increase in profibrotic cytokines in old LN, this suggests that IL‐7 bioavailability may be suboptimal with aging, making LN fibrosis an interesting potential target for both mechanistic and therapeutic studies.

We conclude that restoration of thymic cellularity and of new T‐cell production is not, by itself, sufficient to improve immune protection against intracellular pathogens. Rather, we show that deterioration of SLO in aged animals contribute to the decline of T‐cell maintenance and function, and have the potential to negate the beneficial effects of thymic rejuvenation. These defects, therefore, must be taken into account when considering immune rejuvenation in the old age.

## 4 EXPERIMENTAL PROCEDURES

### 4.1 Animals, KGF, and degarelix treatments

Old (O, 18–22 months of age, male) and adult (A, 2–4 months, male) C57BL/6 (B6) mice were obtained from the National Institute of Aging breeding colony and/or The Jackson Laboratory (Bar Harbor, ME). B6.Rag2pGFP mice were a kind gift from Dr. Michel Nussenzweig (Rockefeller University, New York, NY) via Dr. Pam Fink (University of Washington, Seattle, WA) (Houston, Higdon, & Fink, 2011); they were crossed to B6.SJL‐Ptprc^a^Pepc^b^/BoyJ mice (Ly5.1, CD45.1) and maintained at the University of Arizona vivarium under specific pathogen‐free conditions. B6 and B6.Rag2pGFP‐Ly5.1 mice from 3 to 21 months of age were used to generate a cross‐sectional time course of the decline in naïve T cells. Mice were treated with degarelix (Firmagon) with a single dose of 40 μg per gram of mouse i.p. (Velardi et al., 2014) or with KGF (Palifermin, Kepivance; Amgen, Thousand Oaks, CA) at 5 mg kg^−1^ day^−1^ for 3 consecutive days i.p. (Min et al., 2007). Experiments were conducted under the approval of the Institutional Animal Care and Use Committee (IACUC) and the Institutional Biosafety Committee, in accordance with all applicable federal, state, and local regulations. All WNV experiments were completed within a USDA‐inspected biosafety level 3 facility.

Colony‐bred rhesus macaques (*Macaca mulatta*, RM, Indian origin) of both sexes (two females and one male per group) were maintained according to federal, state, and local guidelines at the Oregon National Primate Research Center under the approval of the Center's IACUC. Old (20–30 years of age) and adult (9–15 years of age) RM were given a low‐dose (250 μg/kg KGF) (Seggewiss et al., 2007) or high‐dose treatments of 1,000 μg/kg or 5,000 μg/kg KGF for 3 consecutive days subcutaneously.

### 4.2 WNV and Lm‐OVA infections

West Nile virus NY 385–99, isolated from the liver of a snowy owl, and in its second passage, was a kind gift from Dr. Robert Tesh (University of Texas Medical Branch at Galveston, Galveston, TX) (Pugh et al., 2016). Virus was prepared as previously described (Uhrlaub et al., 2011). Mice were infected by footpad (f.p.) with 1,000 pfu WNV/50 µl/mouse or i.v. with 10^5^ colony‐forming units of *Listeria monocytogenes* carrying the ovalbumin gene at 42–60 d post rejuvenation treatment. Mice were monitored for survival until 45 days p.i.

### 4.3 Tissue harvest, cell counts, and flow cytometry

Mouse tissues were analyzed 30–45 days post‐treatment or at indicated times following infection. Blood was collected and leukocytes isolated either by hypotonic lysis or by density centrifugation with Lympholyte‐Mammal (Cederlane). Thymus, spleen, and peripheral lymph nodes (one popliteal, inguinal, and brachial were pooled together) were collected and Accutase (Thermo Fisher Scientific)‐digested as previously described (Davies et al., 2017). Tissues were pushed through 40 μm cell strainers. Cell counts were obtained from a Hemavet cell counter (Drew Scientific, Dallas, TX, USA) (Pugh et al., 2016). Cells were stained with a saturating, pretitrated dose of each antibody (>5 μg/ml). Thymocytes were stained with CD44 (IM7), CD117 (ACK2), CD3 (17A2), CD24 (M1/69), CD25 (PC61), NK1.1 (PK136), CD11b (M1/70), Gr‐1 (Ly‐6G), CD19 (eBio1D3), B220 (RA3–6B2), CD4 (RM4–5), and CD8 (53–6.7). Spleen and LN cells were stained for 30 min with antibodies against CD44 (IM7), CD62L (MEL‐14), CD3 (17A2), CD4 (RM4–5), and CD8a (53–6.7) (eBioscience and BioLegend). Cells were then stained with Live/Dead viability dye (Thermo Fisher Scientific). To determine the T‐cell response to WNV or Lm‐OVA, cells were stained overnight with the above antibodies and the H2‐D^b^/NS4b tetramer (NIH Tetramer Facility, Atlanta, GA) and I‐A^b^/E641 tetramer, a gift from Dr. Mike Kuhns (University of Arizona, Tucson, AZ) or with H‐2K^b^‐OVA tetramer, stained for Live/Dead viability, fixed, permeabilized, and stained for intracellular granzyme B (GzB (gb12; Thermo Fisher Scientific). For RM, blood was obtained by venipuncture into heparinized tubes, PBMC isolated by gradient centrifugation (Ficoll‐Hypaque), and stained fresh or cryopreserved as previously described (Okoye et al., 2015). Cells were stained with antibodies against CCR7 (3D12), CD4 (L200), CD95 (DX2), CD28 (CD28.2), CCR5 (3A9), CD3 (SP34–2), CD8a (SK1), and Ki67 (B56) (all from BD Biosciences, Thousand Oaks, CA), as previously described (Okoye et al., 2015). Optimization procedures for flow cytometry are based on current standards and include full‐minus‐one (FMO) gating controls (Roederer, 2002).

Samples (5 × 10^4^ to 1 × 10^6^ cells/sample) were acquired using the Fortessa cytometer (BD Immunocytometry Systems) and then analyzed with FlowJo software (Tree Star).

### 4.4 WNV‐specific IgG ELISA

ELISA was used to determine serum antibody titer against WNV E protein as previously described (Uhrlaub et al., 2011; Widman, Ishikawa, Fayzulin, Bourne, & Mason, 2008).

### 4.5 LN analysis for the presence of fibrosis

LN were immediately fixed in 4% paraformaldehyde/PBS (mouse) or 10% neutral‐buffered formalin (RM) at 4°C overnight, then routinely processed for paraffin embedding, and cut to obtain 5‐μm‐thick sections. Before use, sections were deparaffinized, rehydrated, and incubated with a 0.1% Sirius Red solution (Direct Red 80, Sigma‐Aldrich, St. Louis, MO, USA) in aqueous saturated picric acid, washed in acidified water (0.5% Acetic acid), dehydrated, and mounted with Permount medium. Sections were photomicrographed with a digital camera (Leica DFC450) mounted on light microscope (Leica DMI6000), digitized (LAS X, Inc. software), and analyzed with *ImageJ* software (NIH, Bethesda, MD, USA).

### 4.6 Statistical analysis

Statistics were performed using Prism 7 (GraphPad, San Diego, CA). One‐way and two‐way ANOVA were used to compare groups. Some of the data were not normally distributed as determined by Shapiro–Wilk normality test. In those cases, we ran the Kruskal–Wallis test to confirm significance and it was maintained. Adjusted *p*‐values of <0.05 were considered significant. Nonlinear fit analysis was used to compare whether best‐fit values were shared between data sets. Significance is noted as follows throughout: ns = not significant, *p < 0.05, **p < 0.01, ***p < 0.001, ****p < 0.0001. Error bars denote *SEM*.

## CONFLICT OF INTEREST

L.J.P. Is co‐founder of Vir, Inc. J.N.‐Ž. is co‐chair of the board of Young Blood Institute, Inc. Neither of these entities have had any role in the funding, design, or interpretation of data in this manuscript. Other authors declare no conflict of interest.

## AUTHOR'S CONTRIBUTION

H.L.T. performed degarelix experiments and initial fibrosis analysis in the mouse LN, analyzed data, and wrote the manuscript; M.J.S. performed murine KGF experiments, analyzed data, and wrote the manuscript; J.L.U. performed WNV infections for all animal groups/experiments and wrote the manuscript; I.J. performed advanced analysis of fibrosis in both mice and RM LN; M.J. and S.W. performed select mouse experiments; N.C., A.M.L., and A.O. performed RM KGF experiments; A.O. edited the manuscript; C.D.S. designed the experiments; L.J.P. and J.N.‐Ž. conceived and directed the RM experiments, directed the experiments, and wrote the manuscript. J.N.‐Ž. conceived and directed the mouse experiments.
