# Supplementary figures and images for: Lymph nodes as barriers to T‐cell rejuvenation in aging mice and nonhuman primates
Source: Aging Cell. 2018 Nov 14;18(1):e12865. doi: 10.1111/acel.12865 (PMC6351843; doi:10.1111/acel.12865)

**Figure S1**

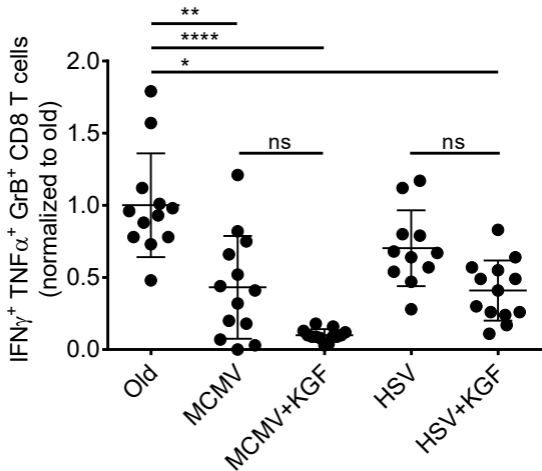

Supplement: Supplementary file 1 [file ACEL-18-e12865-s001.pdf]
